# Getting a Grip on Memory: Unilateral Hand Clenching Alters Episodic Recall

**DOI:** 10.1371/journal.pone.0062474

**Published:** 2013-04-24

**Authors:** Ruth E. Propper, Sean E. McGraw, Tad T. Brunyé, Michael Weiss

**Affiliations:** 1 Department of Psychology, Montclair State University, Montclair, New Jersey, United States of America; 2 Psychology Department, Tufts University, Medford, Massachusetts, United States of America; 3 Development and Engineering Center, US Army Natick Soldier Research, Natick, Massachusetts, United States of America; University of New England, Australia

## Abstract

Unilateral hand clenching increases neuronal activity in the frontal lobe of the contralateral hemisphere. Such hand clenching is also associated with increased experiencing of a given hemisphere’s “mode of processing.” Together, these findings suggest that unilateral hand clenching can be used to test hypotheses concerning the specializations of the cerebral hemispheres during memory encoding and retrieval. We investigated this possibility by testing effects of unilateral hand clenching on episodic memory. The hemispheric Encoding/Retrieval Asymmetry (HERA) model proposes left prefrontal regions are associated with encoding, and right prefrontal regions with retrieval, of episodic memories. It was hypothesized that right hand clenching (left hemisphere activation) pre-encoding, and left hand clenching (right hemisphere activation) pre-recall, would result in superior memory. Results supported the HERA model. Also supported was that simple unilateral hand clenching can be used as a means by which the functional specializations of the cerebral hemispheres can be investigated in intact humans.

## Introduction

Simple clenching of one versus the other hand increases the neuronal activity of the frontal lobe in the opposite (contralateral) hemisphere [1], [2]. Electroencephalographic (EEG) measures demonstrate that a mere 90 seconds of left hand clenching increases right hemisphere activity, and similar right hand clenching increases left hemisphere activity [1].

This form of hand clenching has also been associated with increased experiencing of a given hemisphere’s emotional mode of processing. Specifically, right hand clenching (left hemisphere activation) versus left hand clenching (right hemisphere activation) results in increased approach (e.g.: happiness, anger) versus withdrawal (e.g.: sadness, anxiety) emotional states, respectively [1], [2], [3], [4]. Given that the left prefrontal cortex is associated with the experiencing of approach emotions, and the right prefrontal cortex with withdrawal emotions [5], [6], [7], these results indicate that sustained unilateral hand clenching, by differentially activating one versus the other hemisphere, increases the experiencing of processes associated with the more active hemisphere.

The above offers the tantalizing possibilities that unilateral hand clenching i) may be a viable – and novel - method by which the specializations of the cerebral hemispheres can be investigated in intact humans, and ii) may increase performance on tasks differentially demanding of one versus the other hemisphere’s neural resources. Given that the two cerebral hemispheres are thought to be differentially involved in many functions, including language, emotion, spatial processing, and local/global information [8], unilateral hand clenching may be beneficial in investigations ranging from those of basic research to clinical applications.

The effects of unilateral hand clenching on emotional/motivational state have been documented [1], [2], and at least one other study has manipulated hemispheric activation via unilateral hand clenching in order to test hypotheses related to asymmetrical hemispheric contributions to perceptual processing [9]. However, to our knowledge no research has examined cognition, and in particular memory processing, as a function of hemispheric activation induced via unilateral hand clenching.

The Hemispheric Encoding/Retrieval Asymmetry (HERA) model proposes that left prefrontal regions are associated with encoding, and right prefrontal regions with retrieval, of episodic memories [10], [11]. Because increased activity of a given hemisphere is associated with an increase in that hemisphere’s mode of processing (i.e.; the line of reasoning outlined above), increasing one versus the other hemisphere’s neuronal activity immediately prior to encoding, and immediately prior to recalling information, should influence recall ability, in the context of the HERA model. Given the relationship between hand clenching and cortical activity, in conjunction with the HERA model’s proposed differential involvement of the left versus right hemispheres in encoding versus retrieval of episodic information, respectively, it was hypothesized that right hand clenching (left hemisphere activation) prior to encoding, and left hand clenching (right hemisphere activation) prior to recall, would result in superior recall for episodic information.

## Materials and Methods

### Ethics Statement

The research was approved by the Montclair State University IRB and the U.S. Army Human Research Protection Office. Participants provided their written informed consent to participate in the study.

### Participants

51 right-handed individuals (score of +80 or higher on the Edinburgh Handedness Inventory [12]), participated as part of a larger study. (Given known differences in functional and structural cortical organization between right- and non-right-handers [15], [16], and given the known superiority of non-right-handers on episodic memory tasks [17], [18], only right-handed individuals were analyzed here. Left-handed [−80 and below, n = 4], and inconsistently-handed (between +/−80, n = 94) individuals were also tested. Results for these other two handedness groups will be reported elsewhere.) Participants received either Psychology course credit or $20.00 remuneration for their participation. One participant was eliminated from analyses due to illegible handwriting (final N = 50; 40 women). Age ranged from 18 to 48 years, with a mean of 23.31 years (*SE* = 1.07; one participant’s age was not recorded, and thus ages are based on N = 49).

Participants were randomly assigned to one of five Hand Clench Conditions, with participants clenching their left or right hand pre-encoding (Lenc or Renc, respectively) and their left or right hand pre-recall (Lrec or Rrec, respectively). An additional no clenching control group did not clench either hand pre-encoding or pre-recall (See Table 1 for group ns). (We also tested eleven other conditions [N = 103 right-handers]. These included pre-encoding left or right clenching and pre-recall no clenching; pre-encoding no clenching, and pre-recall left or right clenching; pre-encoding bilateral [both hands clenching simultaneously] clenching, and pre-recall left, right, no clenching or bilateral clenching; and pre-encoding left, right, or no clenching, and pre-recall bilateral clenching. For clarity we are focusing only on the critical comparisons listed in the Methods section, above, here. Additional analyses on the other Hand Clenching Conditions will be reported elsewhere.).

**Table 1:** Means (Standard Errors) of the Dependent Measures as a Function of Hand Clench Condition.

Measure					
Hand Clench Condition	n	Total Written	Hits	False Alarms	Correct Scores
**Lenc/Lrec**	9 (6 women)	6.67 (.82)	5.67 (.64)	1.00 (.41)	4.67 (.71)
**Renc/Rrec**	11 (10 women)	8.00 (.73)	7.54 (.62)	.46 (.16)	7.09 (.55)
**Lenc/Rrec**	11 (8 women)	7.00 (.81)	6.18 (.64)	.82 (.42)	5.36 (.73)
**Renc/Lrec**	9 (9 women)	11.11 (1.56)	10.11 (1.75)	1.00 (.44)	9.11 (2.02)
**NENR**	10 (7 women)	9.60 (1.33)	8.60 (1.33)	1.00 (.39)	7.60 (1.44)

### Materials

#### Memory stimuli

72 words randomly taken from [13] were used to create two lists of 36 words (two lists were created for counterbalancing purposes).

#### Clenching stimuli

Participants were instructed via computer and by the experimenter to squeeze a pink, 5 cm diameter, rubber ball as hard as possible for two sets of 45 seconds, with an intervening 15 second break, per pre-encoding and pre-recall condition. A no clenching control group held the same ball gently in both hands.

### Procedure

Participants were tested individually, and word stimuli and all instructions were presented via Superlab v. 4.5. Following consent procedures, participants completed pre-encoding clenching. For both pre-encoding and pre-recall clenching, participants were instructed to focus on an ‘X’ in the center of the computer screen. Participants were instructed to squeeze the rubber ball in their left or right hand ‘as hard as they could’ for 45 seconds while looking straight ahead at the ‘X’. Following a 15 second rest, participants squeezed again for 45 seconds. Initiation and termination of squeezing was indicated by brief (two second) tones, and by concurrent experimenter instructions. Participants in the no-clenching condition were instructed to hold a rubber ball in cupped hands while they focused on the ‘X’. When the tones occurred in this condition, participants were instructed to ‘continue holding the ball gently’.

Immediately following pre-clenching condition, participants’ ear temperatures were taken (to be reported elsewhere), participants then were presented with the list words and asked to ‘study the words because they will be tested on them later’. List words were presented at the rate of 5 seconds each, in upper case, 28 point, Courier New font on a 21.5 inch iMac computer monitor. Following word presentation, participants completed the Edinburgh Handedness Inventory [12], and a filler questionnaire (Waterloo Handedness Questionnaire). Next, participants engaged in pre-recall clenching (or no-clenching), wherein the procedures were identical to that of pre-encoding clenching, and were followed immediately by ear temperature measurements. Participants were then asked to recall as many words from the list they saw earlier as they could using paper and pencil.

## Results

One-way analyses of variance (ANOVA; Hand Clench Condition: Right Encoding/Right Recall [Renc/Rrec] vs Right Encoding/Left Recall [Renc/Lrec] vs Left Encoding/Left Recall [Lenc/Lrec] vs Left Encoding/Right Recall [Lenc/Rrec] vs None Encoding/None Recall [NENR])) were conducted on the total number of words written, total number of words correctly recalled (hits), total number of items ‘recalled’ that had not been presented (false alarms), and corrected score (hits minus false alarms [14].

**Figure 1:**
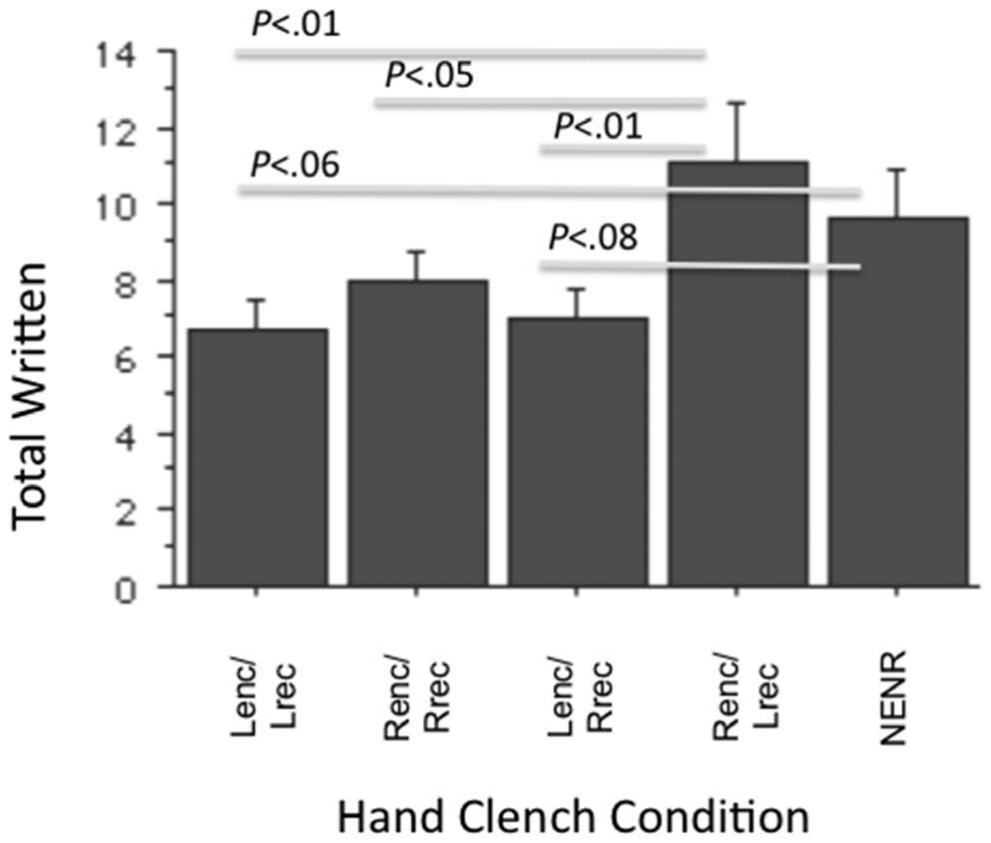
Total written as a function of hand clench condition.

The ANOVA examining total written was significant (*F*(4, 45) = 2.88, *p*<.05). *Post hoc* examination of simple effects (Fisher’s PLSD) revealed that Renc/Lrec was greater than Lenc/Lrec (*p*<.01, *d* = 1.19), greater than Renc/Rrec (*p*<.05, *d* = .84), and greater than Lenc/Rrec (*p*<.01, *d* = 1.08). There were strong trends for NENR to be greater than Lenc/Lrec (*p* = .06, *d* = .85) and Lenc/Rrec (*p* = .08, *d* = .74). See Figure 1.

**Figure 2:**
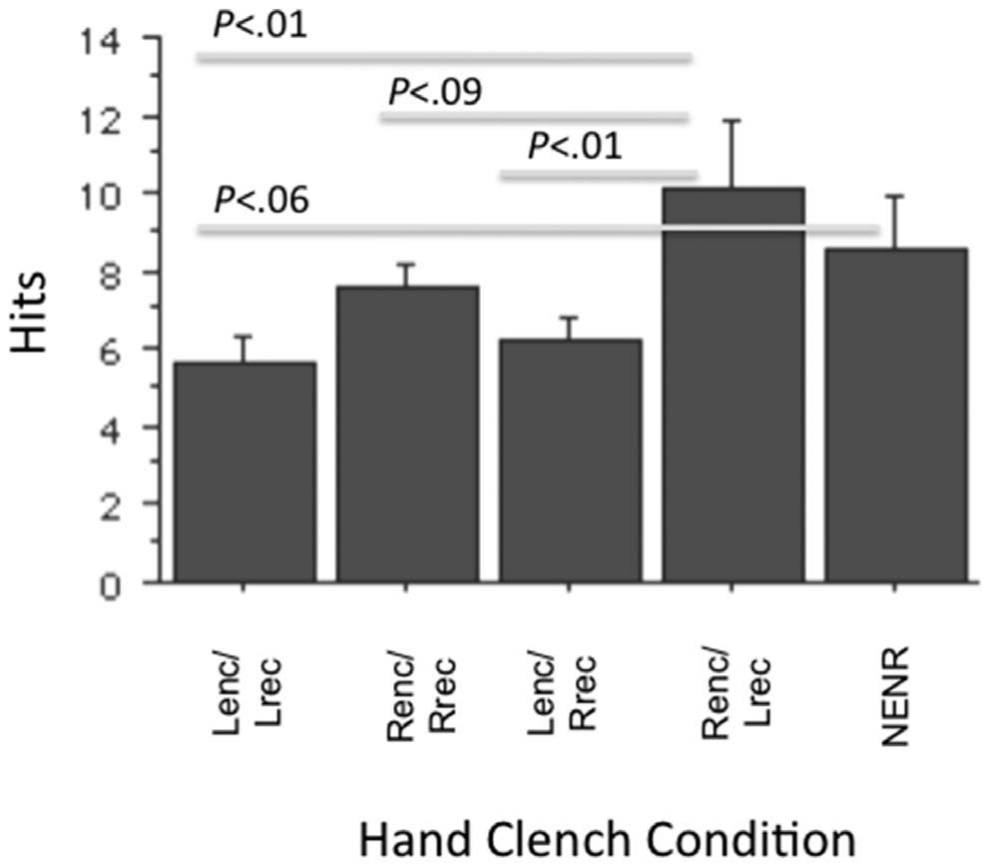
Hits as a function of hand clench condition.

The ANOVA examining hits was also significant (*F*(4,45) = 2.77, p<.05). *Post hoc* examination of simple effects (Fisher’s PLSD) were similar to those of total written, with those in the Renc/Lrec condition outscoring the Lenc/Lrec (*p*<.01, *d* = 1.12) and Lenc/Rrec (*p* = .01, *d = *.98) conditions, and a strong trend to outscore the Renc/Rrec (*p* = .09, *d* = .64) as well. The NENR demonstrated a strong trend to recall more items than the Lenc/Lrec (*p* = .06, *d* = .90). See Figure 2.

The ANOVA examining false alarms did not attain significance (p>.5), nor did any simple effects.

**Figure 3:**
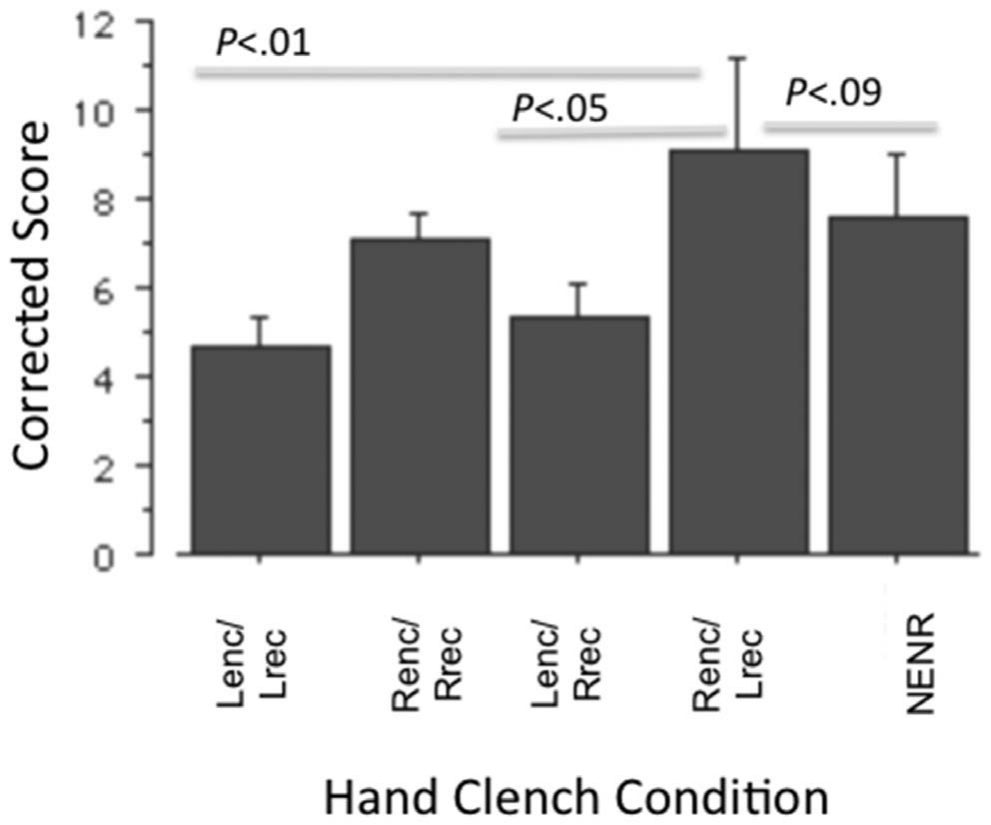
Corrected scores as a function of hand clench condition.

The ANOVA examining corrected scores did not reach traditional significance (*F*(4,45) = 2.20, *p* = .08), but *post hoc* analyses of simple effects (Fisher’s PLSD) revealed results that mirrored the results above, with Renc/Lrec scoring greater than Lenc/Lrec (*p* = .01, *d* = .98) and Lenc/Rrec (*p*<.05. *d* = .81), and a trend for the NENR to score higher than the Lenc/Lrec (*p* = .09, *d* = .82). See Figure 3.

See Table 1 for means and standard errors of the dependent measures as a function of Hand Clench Condition.

## Discussion

Individuals who encoded language-based information immediately following right hand clenching (left hemisphere activation), and recalled such information immediately following left hand clenching (right hemisphere activation), demonstrated superior episodic memory compared to the other hand clenching conditions. It is noteworthy that this condition was also superior to the no hand clenching control condition, though not significantly so. This may have been due to the small sample sizes, and slightly increased variability in these two comparison groups (See Table 1). It may be that increasing sample sizes would result in a significant difference between the Renc/Lrec and NENR groups.

The other three hand clenching conditions did not differ from each other; however, left hand clenching (right hemisphere activation) pre-encoding, and right hand clenching (left hemisphere activation) pre- recall, resulted in significantly poorer memory compared to the no hand clenching condition, as did the left hand clenching (right hemisphere activation) in both the pre-encoding and pre-recall conditions. Together, this pattern of results i) supports the HERA model’s prediction of left hemisphere encoding/right hemisphere retrieval of episodic information [10], [11], and ii) suggests that it is primarily the hemisphere active at encoding that predominantly influences memory ability. This latter is indicated because, although the two pre-encoding left hand clenching (right hemisphere activation) conditions, regardless of Hand Clench Condition at pre-recall, demonstrated significantly poorer recall than the no clenching control condition, the two right hand clenching (left hemisphere activation) pre-encoding conditions did not (regardless of Hand Clench Condition at pre-recall). In fact the right clenching pre-encoding/left clench pre-recall condition was numerically greater than the no clenching condition.

It is not clear why the Hand Clench Conditions did not differ in the number of false alarms. One possibility is a floor effect in the number of falsely recalled words; future studies could increase the amount of time elapsing between encoding and recall, or by manipulating the encouragement of ‘guesses’ to further investigate this issue. Likewise, given the right hemisphere’s involvement in spatial processing, it would be useful to investigate whether the left hemisphere’s superiority at encoding would remain when information to be recalled is spatially-based.

We did not measure hemispheric activation directly in the current study. However, previous work [1], [2] demonstrating that identical hand clenching activates the contralateral prefrontal cortex suggests that this mechanism accounts for the results presented here. Future work could directly measure hemispheric activity and memory following hand clenching in order to confirm that increased hemispheric activity following hand clenching is in fact the mechanism of action for the effects. Additionally, we would like to point out that the stimuli used here were language-based. It not known whether pictorial or spatially-based stimuli would also benefit from Renc/Lrec. The HERA model predicts left hemisphere encoding, and right hemisphere retrieval, of episodic information, regardless of stimuli type. Future research could examine this hypothesis directly [10], [11].

In total, these results are striking, given that the manipulation used- a total of 90 seconds of unilateral hand clenching pre-encoding and pre-recall- is easily adaptable to a variety of experimental, clinical, and real-world situations. Additionally notable is that the sizes of the effects (*d*) tended to be large or very large, with only two comparisons being in the medium range, supporting the robustness of the findings. The findings presented here offer the exciting possibility that simple unilateral hand clenching can be used as a means by which the functional specialization of the cerebral hemispheres can be investigated and possibly adapted to practical situations.
