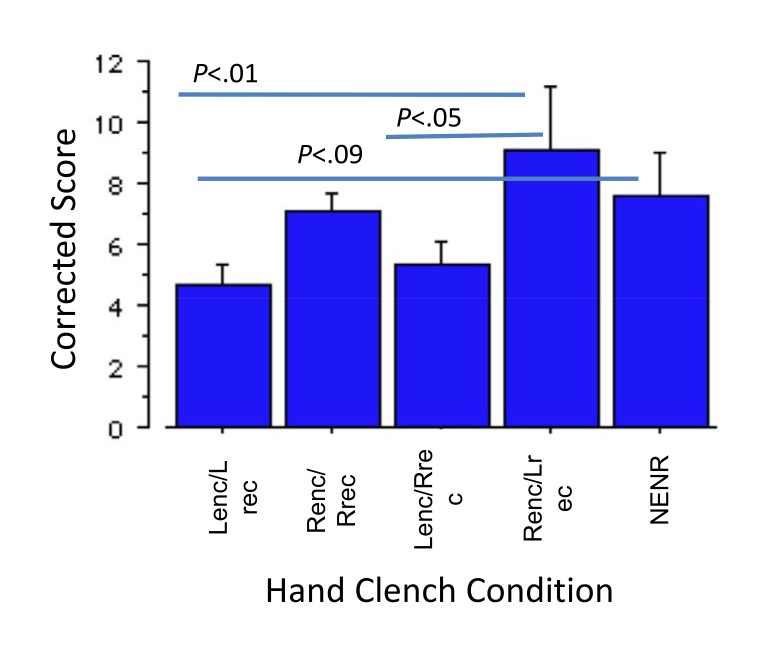# Correction: Getting a Grip on Memory: Unilateral Hand Clenching Alters Episodic Recall

**DOI:** 10.1371/annotation/693ff849-c230-4e59-8772-b8755daf0f75

**Published:** 2013-05-03

**Authors:** Ruth E. Propper, Sean E. McGraw, Tad T. Brunyé, Michael Weiss

There was an error in Figure 3. The revised version can be viewed here: 

**Figure:**